# Contribution of miR-124 rs531564 polymorphism to the occurrence of congenital Zika syndrome

**DOI:** 10.1080/15592294.2022.2145061

**Published:** 2022-11-21

**Authors:** Julia A Gomes, Igor Araujo Vieira, Eduarda Sgarioni, Ana Cláudia P Terças-Tretell, Juliana H da Silva, Bethânia FR Ribeiro, Marcial F Galera, Thalita M de Oliveira, Maria Denise F Carvalho de Andrade, Isabella F Carvalho, Lavínia Schüler-Faccini, Fernanda SL Vianna

**Affiliations:** a Programa de Pós-Graduação em Genética e Biologia Molecular (PPGBM), Departamento de Genética, Universidade Federal do Rio Grande do Sul (UFRGS), Porto Alegre, Brazil; b Sistema Nacional de Informação sobre Agentes Teratogênicos (SIAT), Serviço de Genética Médica (SGM), Hospital de Clínicas de Porto Alegre (HCPA), Porto Alegre, Brazil; c Instituto Nacional de Genética Médica Populacional (INAGEMP), Hospital de Clínicas de Porto Alegre (HCPA), Porto Alegre, Brazil; d Laboratório de Medicina Genômica (LMG), Centro de Pesquisa Experimental (CPE), Hospital de Clínicas de Porto Alegre (HCPA), Porto Alegre, Brazil; e Departamento de Enfermagem, Universidade do Estado de Mato Grosso (UNEMAT), Tangará da Serra, Brazil; f Programa de Pós-graduação em Saúde Coletiva, Universidade Federal de Mato Grosso (UFMT), Cuiabá, Brazil; g Secretaria Municipal de Saúde de Tangará da Serra, Tangará da Serra, Brazil; h Fundação Hospital de Clínicas Do Acre (FUNDACRE), Rio Branco, Brazil; i Departamento de Pediatria, Faculdade de Medicina, Universidade Federal de Mato Grosso (UFMT), Cuiabá, Brazil; j Hospital Universitário Júlio Müller (HUJM), Universidade Federal de Mato Grosso (UFMT), Empresa Brasileira de Serviços Hospitalares (EBSERH), Cuiabá, Brazil; k Universidade Estadual do Ceará (UECE), Fortaleza, Brazil; l Centro Universitário Christus (UNICHRISTUS), Fortaleza, Brazil; m Escola de Saúde, Universidade do Vale do Rio do Sinos (Unisinos), São Leopoldo, Brazil

**Keywords:** Maternal exposure, zika virus infection, congenital abnormalities, gene expression, genetic variation, transferrin receptor, microRNAs, disease susceptibility

## Abstract

Zika virus (ZIKV) cause Congenital Zika Syndrome (CZS) in individuals exposed during pregnancy. Studies have shown that ZIKV infection positively regulates the miR-124 expression in neural cells, which leads to a decrease of TFRC, a gene targeted of this miRNA. Both miR-124 and TFRC exhibit a pivotal role in nervous system development. Therefore, in this study we aimed to investigate whether genetic variants that affect the expression of these genes could act together with ZIKV to increase the risk of individuals developing CZS. TFRC rs406271 and MIR-124-1 rs531564 polymorphisms were genotyped, using TaqMan® Genotyping Assays, in a sample of children who were exposed to ZIKV during pregnancy, of whom 40 were born with CZS and 48 without congenital anomalies. We identified that individuals with CZS presented a higher frequency of CG genotype of rs531564 polymorphism in MIR-124-1 (p=0.048), which is associated with increased expression of miR-124. Since ZIKV also upregulates the expression of this miRNA, the presence of CG genotype in individuals exposed to the virus could lead to a scenario of overexpression of miR-124 in the brain. Since teratogenesis is a multifactorial event, this genetic finding could partly explain why such individuals are more susceptible to CZS, considering both the downregulation of important neurodevelopment genes, as well as deregulation of the neurogenesis process. Thus, we provide preliminary evidence about a possible genetic risk factor to CZS and highlight the importance of analyzing functional polymorphisms related to epigenetic modulators of neurodevelopment genes in the context of ZIKV teratogenesis.

## Introduction

Congenital Zika syndrome (CZS) is a set of congenital anomalies developed due to exposure of the embryo or foetus to Zika virus infection (ZIKV) during pregnancy (Del Campo et al., 2017). Although the symptoms of infection in mothers are mild, or even non-existent (Duffy et al., 2009), the consequences of the congenital infection can be severe. The phenotypic spectrum of CZS includes mainly brain malformations, such as microcephaly, ventriculomegaly, intracranial calcifications, cerebral atrophy, and lissencephaly, as well as ocular and neuropsychomotor alterations (Del Campo et al., 2017).

It is estimated that only about 30–42% of cases exposed to the congenital infection develop any type of congenital anomaly (Pomar et al., 2019; Vhp et al., 2020), demonstrating a differential susceptibility to the development of CZS. Studies with animal models have shown, for example, that inefficient binding of the ZIKV NS5 protein to mouse Stat2 renders the virus unable to antagonize the mouse IFN signalling, which ensures the species’ resistance to the infection and adverse outcomes (Grant et al., 2016; Gorman et al., 2018). On the other hand, genetic susceptibility to CZS in humans has been investigated through the presence of pathogenic variants in genes related to CZS phenotypes, or related to neurodevelopment and immune response pathways in affected individuals (Caires-Júnior et al., 2018; Aguiar et al., 2020; Borda et al., 2021; Gomes et al., 2021).

In order to understand how congenital infection impairs neurodevelopment, some researchers have investigated the molecular changes induced by ZIKV in neural cells (Rolfe et al., 2016; Caires-Júnior et al., 2018). In their studies, infection in neural cells has been shown to disrupt the expression of several microRNAs (miRNAs) (Azouz et al., 2019; Dang et al., 2019), which are post-transcriptional regulators, acting through degradation and/or translational repression of the messenger RNAs (mRNAs) of their target genes (Catalanotto et al., 2016). One of these miRNAs is miR-124, which seems to be upregulated in human neuronal stem cells (hNSC) infected by ZIKV (Azouz et al., 2019; Dang et al., 2019).

miR-124, encoded by the *MIR-124-1* gene, is generally expressed concomitantly with the neurogenesis process. It is progressively upregulated during the central nervous system (CNS) development, where it is involved in a wide spectrum of biological functions, such as the transition from neural progenitors to neurons (Maiorano & Mallamaci, 2009; Sun et al., 2015). Overexpression of miR-124 has been shown to accelerate neuronal differentiation and its inhibition induces cell cycle arrest in neural progenitors (Coolen & Bally-Cuif, 2009; Sun et al., 2015). Therefore, for neurogenesis to occur successfully, there is a refined control of miR-124 expression and its deregulation contributes to CNS disorders (Papagiannakopoulos & Kosik, 2009; Sun et al., 2015).

Dang and colleagues (2019) suggest that miR-124 may be involved in the ZIKV-induced microcephaly phenotype due to the deregulation of the expression of its target genes (Dang et al., 2019). The authors show the transferrin receptor-related mRNA (*TRFC*) to be a target of this miRNA, and during ZIKV infection it negatively regulates *TFRC* levels in hNSC-derived neurospheres, resulting in neurosphere size reduction, which may be involved in the microcephaly phenotype (Dang et al., 2019). *TRFC* encodes the transferrin receptor TFR1 that mediates cellular iron uptake (Gammella et al., 2017). In addition to regulating iron uptake and metabolism, it plays a role in nervous system development, stem cell renewal and cell cycle regulation (Levy et al., 1999; Silvestroff et al., 2013; Gammella et al., 2017).

Given that not all individuals exposed to congenital ZIKV infection develop CZS, factors associated with this differential susceptibility must be investigated. It should be noted that genetic susceptibility partially explains the effects of some teratogenic agents that affect the development of embryos and foetuses (Cassina et al., 2012). Therefore, since ZIKV alters the expression of miR-124 and *TFRC*, we wondered whether genetic variants present in these genes, which also modulate their expression, could act on the susceptibility to CZS. To answer this question, in this study we evaluate the role of functional genetic variants within *MIR-124-1* and *TRFC* genes as susceptibility factors to CZS in a sample of children exposed to ZIKV during their intra-utero life, some of whom developed CZS while others were born without congenital anomalies.

## Methodology

### Ethical issues

This study was carried out following the rules of the Declaration of Helsinki and was approved by the Ethics and Research Committee of Hospital de Clínicas de Porto Alegre, the institution responsible for this study (n° 170,619 – CAAE 78735817910015327), and by all participating institutions. All legal guardians of individuals recruited for this study gave their informed consent for inclusion before they participated in the study.

### Sample

In this case-control study we included 40 children diagnosed with CZS whose mothers had evidence of ZIKV infection during pregnancy (case group) and 48 children without congenital anomalies whose mothers also had evidence of ZIKV infection (control group). Evidence of ZIKV exposure was defined as positive RT-PCR or clinical-epidemiological criteria, i.e., specific symptoms of ZIKV infection during a ZIKV outbreak in the region during pregnancy (e.g., rash, fever and/or joint pain). Case children were recruited between June 2018 and November 2019 from reports of microcephaly in five Brazilian research and/or healthcare centres: North region (Fundação Hospital do Acre, n = 4), Northeast region (Fundação Universidade Estadual do Ceará, n = 21), Midwest region (Universidade do Estado de Mato Grosso, n = 2 and Universitário Júlio Muller, n = 12) and South region (Hospital de Clínicas de Porto Alegre, n = 1). Control children were recruited in the same research and/or healthcare centres from the North region (n = 1), Midwest region (n = 46, from a cohort of women that gave birth in 2016, in the city of Tangará da Serra) and South region (n = 1). Clinical and sociodemographic characteristics were obtained through medical records and questionnaires administered during the medical consultation.

### Genetic analysis

A blood or saliva sample was collected from individuals and DNA extraction was performed by the FlexiGene DNA Kit (Qiagen®) or Oragene Kit (DNA Genotek), respectively. Polymorphism in *TFRC* (rs406271 – TaqMan assay C_976917_20) and in *MIR-124-1* (rs531564 – TaqMan assay C_772662_10) genes was genotyped through the TaqMan® Genotyping Assay method in Step One PlusTM Real-Time PCR Systems.

The criteria to select such polymorphisms involved their Minor Allele Frequency (MAF) > 1% (based on gnomAD database information for non-Finnish European population and AbraOM database for Brazilian population) and their functional description as modifiers of their gene expression (Qi et al., 2012; Serre et al., 2008).

### Statistical analyses

A descriptive analysis of the sample was performed using frequencies or proportions, measures of central tendency and dispersion. The Hardy-Weinberg equilibrium was tested for both polymorphisms. Categorical variables were compared between the groups by chi-square test or Fisher’s Exact Test. P values lower than 0.05 were considered to be significant. SPSS® v.18 software was used to perform the statistical analyses.

## Results

Eighty-eight children exposed to ZIKV during their intra-utero life were recruited for this study. Forty children developed CZS (case group) and 48 were born without congenital anomalies (control group). The complete description of this sample regarding clinical, gestational and sociodemographic aspects was presented in a previous study of our research group (Gomes et al., 2021). A brief description of the sample is also presented in Supplementary Table 1. A higher prevalence of black individuals was found in both case (77%) and control (65%) groups. In addition, mothers of children with CZS presented lower educational level (*p* < 0.001) and lower monthly family income (*p* = 0.008). For the gestational and clinical characteristics, we found that mothers of children with CZS were exposed to ZIKV infection mostly in the first trimester of pregnancy (80%) while mothers of children without CZS were exposed mostly in the third trimester (44%) (*p* < 0.001). As expected, children of the control group presented higher weight, height and cephalic perimeter measurements (*p* < 0.001).

Regarding the congenital anomalies of the children with CZS, they were also described in a previous study of our group (Gomes et al., 2021). In this study they are presented in Supplementary Table 2. The most prevalent clinical findings were microcephaly (confirmed in all children), calcifications, cerebral atrophy, lissencephaly, ventriculomegaly and ocular alterations.

The allelic and genotypic frequencies of polymorphisms in *TFRC* and *MIR-124-1* in both groups are presented in Table 1. The genotypic frequencies of all genetic variants were consistent with the Hardy-Weinberg equilibrium. Regarding the allelic frequencies of *TFRC* rs406271 polymorphism in cases and controls, they were similar to those of the gnomAD (European population) and ABraOM (Brazilian population) databases. Comparing cases and controls, such frequencies did not present statistically significant differences. Likewise, the genotypic frequencies of this polymorphism did not show statistically significant differences between the groups. The allelic frequencies of *MIR-124-1* rs531564 polymorphism were also similar to the gnomAD and ABraOM bases and showed no statistically significant differences. However, interestingly, the genotypic frequencies of this polymorphism showed a statistically significant difference between groups (p = 0.048), with a higher frequency of the CG genotype in cases (35%) compared with controls (17%).

**Table 1.** Allelic and genotypic frequencies of polymorphisms in *TFRC* and *MIR-124-1* genes in the case and control groups.

Gene	SNP	Allele/Genotype	gnomAD	ABraOM		Case (n = 40)	Control (n = 48)	*p-*value†
*TFRC*	rs406271	T				60 (75%)	64 (67%)	0.228
(n, %)		C	32%	32%		20 (25%)	32 (33%)	
		TT				23 (57%)	22 (46%)	0.570
		CT				14 (35%)	20 (42%)	
		CC				3 (8%)	6 (12%)	
*MIR-124-1*	rs531564	G				66 (82%)	88 (92%)	0.067
(n, %)		C	14%	12%		14 (18%)	8 (8%)	
		GG				26 (65%)	40 (83%)	0.048*
		CG				14 (35%)	8 (17%)	

gnomAD: allelic frequencies considering the world population from the genome aggregation database (gnomAD); ABraOM: allelic frequencies considering the Brazilian population from Arquivo Brasileiro Online de Mutações database. †Chi-squared test or Fisher’s exact test; *Statistically significant. SNP, single nucleotide polymorphism; TRFC, transferrin receptor protein 1; MIR-124-1, microRNA-124-1.

The allelic and genotypic frequencies of polymorphisms in TFRC and MIR-124-1 were also compared between cases with CZS considering their specific characteristics (sex, ethnicity, trimester of exposure to ZIKV infection and presence of isolated or multiple congenital anomalies) (Supplementary Table 3). Nonetheless, no statistically significant difference was found in these comparisons.

### Discussion

Congenital ZIKV infection was recognized in 2016 as a teratogenic event, that is, capable of affecting the development of foetuses and embryos by causing congenital anomalies. However, it has been reported over the years that, despite the severe consequences of the congenital infection, some exposed individuals did not develop any alteration (Caires-Júnior et al., 2018; Pomar et al., 2019; Vhp et al., 2020). This suggests that genetic risk factors may act on this differential susceptibility for the development of CZS. In this sense, evaluating genetic variants that could act together with ZIKV, affecting gene and protein expression, can help us understand the role of genetics in the occurrence of CZS. Since molecular analyses suggested that ZIKV could cause CZS by inducing a deregulation of miR-124 levels and its target *TFRC* (Azouz et al., 2019; Dang et al., 2019), the aim of this study was to evaluate whether genetic variants that affect the expression of such genes could act together with ZIKV, and, consequently, as risk factors for CZS.

**Figure 1.**
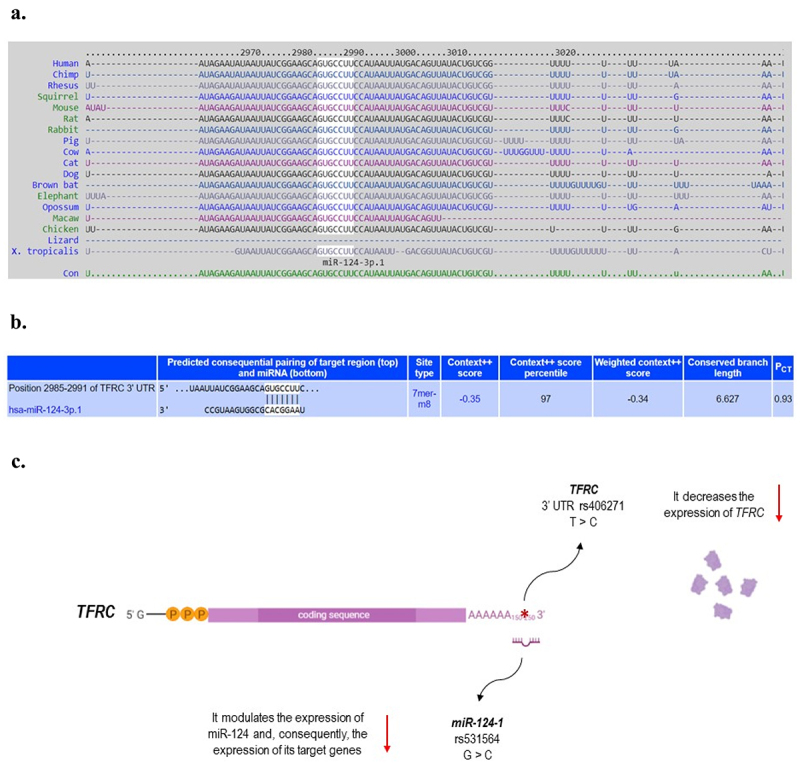
**a**. The white highlighted sequence shows the corresponding perfect base pairing site between miR-124-1 and TFRC 3’ untranslated region (UTR) mRNA, named seed sequence. This sequence in the miRNA binding site at TFRC 3’UTR is highly conserved among different species. **b**. Predicted seed pairing of miR-124-1 with the target region on TFRC 3’UTR. **c**. Functional role of polymorphisms in TFRC and MIR-124-1 included in this study. References: TargetScanHuman database 7.2 (https://bit.ly/3ttzjpk); Ensembl (https://bit.ly/3o1utP0; https://bit.ly/3tD2ma5); (Serre et al., 2008); (Qi et al., 2012). Created with BioRender.com.

As previously mentioned, ZIKV infection has been shown to increase miR-124 expression (Azouz et al., 2019; Dang et al., 2019), leading to downregulation of its target, *TFRC* gene (Dang et al., 2019). Both *MIR-124-1* and *TFRC* are expressed in the brain and act in the context of neurodevelopment (Coolen & Bally-Cuif, 2009; Hänninen et al., 2009; Maiorano & Mallamaci, 2009; Silvestroff et al., 2013; Sun et al., 2015). Since this miRNA has several other targets, also important to neurogenesis and neural differentiation (e.g., *Sox9, JAG1, DLX2, STAT3, LAMC1, ITGB1*, among others) (Coolen & Bally-Cuif, 2009; Sun et al., 2015), its overexpression has been described as impairing these processes (Papagiannakopoulos & Kosik, 2009; Sun et al., 2015). Figure 1 shows, based on studies in the literature, the 3’UTR sequence of *TFRC* that is targeted by miR-124 and how the polymorphisms evaluated in this study affect the expression of the *MIR-124-1* and *TFRC* genes. In this study we identified that individuals with CZS have a higher frequency of the CG genotype for rs531564 polymorphism in *MIR-124-1*. This genotype has been described to increase the expression levels of mature miR-124-1 compared to the GG and CC genotypes (Qi et al., 2012). Therefore, we highlighted, for the first time, that the effect of ZIKV increasing the expression of this miRNA, together with the presence of its CG genotype in individuals exposed to the virus, could lead them to a scenario of overexpression of miR-124 in the brain. This could partly explain why such individuals are more susceptible to CZS, due to changes in the gene expression of these miRNA targets, such as *TFRC*, among others mentioned above, involved in the neurogenesis process.

Since little is known about the role of genetics in susceptibility to ZIKV teratogenesis, we believe that studies that assess the genetic variation of individuals exposed to ZIKV during pregnancy could provide interesting insights regarding molecular mechanisms of ZIKV infections and CZS. However, it is important to consider some limitations of this study. First, our relatively small sample size (n = 88 individuals) may have influenced our power to identify stronger associations between the analysed variants and the development of CZS, as well as the reliability of the association identified. In this sense, it is important highlight that the ZIKV epidemic in Brazil occurred between 2015 and 2016, and since 2017 the number of new cases has decreased considerably in the country (Brasil, 2021). In addition to this issue, some characteristics make our sample heterogeneous, such as the trimester of exposure to ZIKV and the origin of the individuals, which could affect both the identification of significant associations in the genotype-phenotype correlation and the interpretation of the associations found. Finally, we emphasize that, unfortunately, the collection of RNA for gene expression evaluation from children with and without CZS was not performed at an opportune time, such as at birth. Thus, we could not compare *TFRC* and *MIR-124-1* gene expression among genotypes. A current sample collection for an expression study would probably not bring interesting results since such gene expression is not high in saliva or blood samples, only in brain cells.

Given the impact that ZIKV infection has on certain miRNAs, modulating the expression of genes that act in neurodevelopment, the investigation of functional genetic variants within their genes, as well as in the 3’UTR of their target genes, is a promising strategy to identify susceptibility factors to ZIKV teratogenesis. In this study, we identified that the CG genotype of rs531564, located in the gene encoding miR-124-1, could represent a risk factor for CZS, suggesting an important role of germline variants acting together with ZIKV in the epigenetic control of specific targets. Clearly, it is fundamental to highlight that studies with larger sample sizes must be performed in order to confirm this result. Finally, our study shows that to evaluate genetic variants in other miRNAs and epigenetic players could be a great strategy to understand individual susceptibility to CZS and molecular pathways of ZIKV-mediated teratogenesis.

## Supplementary Material

Supplemental Material
