# Supplementary material for: Contribution of miR-124 rs531564 polymorphism to the occurrence of congenital Zika syndrome
Source: Epigenetics. 2022 Nov 21;18(1):2145061. doi: 10.1080/15592294.2022.2145061 (PMC9980461; doi:10.1080/15592294.2022.2145061)
Supplement: Supplemental Material [file KEPI_A_2145061_SM1384.docx]

| **Supplementary Table 1**: Clinical, gestational, and sociodemographic characteristics of the case and control groups. | | | |
| --- | --- | --- | --- |
| **Characteristics** | **Cases^a^ (n = 40)** | **Controls (n = 48)** | ***p-*value** |
| Sex (n, %) |  |  |  |
| *Male* | 23 (57%) | 26 (54%) | 0.754^b^ |
| *Female* | 17 (43%) | 22 (46%) |  |
| Ethnicity (n, %) |  |  |  |
| *Black* | 31 (77%) | 31 (65%) | 0.242^b^ |
| *White* | 9 (23%) | 17 (35%) |  |
| Weight (kg) | 2.5 (2.2 – 2.9) | 3.2 (2.8 – 3.5) | **<0.001^c^** |
| Height (cm) | 45.0 (44.0 – 48.0) | 49.0 (47.0 – 50.0) | **0.001^c^** |
| Cephalic perimeter (cm) | 29.0 (27.3 – 31.0) | 35.0 (34.0 – 36.0) | **<0.001^c^** |
| Gestational age at birth (weeks) | 38.0 (37.0 – 39.0) | 38.0 (37.0 – 38.7) | 0.522^c^ |
| Mother’s age (years) | 28.0 (22.5 - 35.5) | 29.5 (22.0 - 33.0) | 0.824^c^ |
| Father’s age (years) | 28.0 (24.0 - 37.8) | 31.0 (26.0 - 35.0) | 0.693^c^ |
| Trimester of ZIKV infection (n, %) |  |  |  |
| *1st* | 24/30 (80%) | 13 (27%) | **<0.001^b^** |
| *2nd* | 4/30 (13%) | 14 (29%) |  |
| *3rd* | 2/30 (7%) | 21 (44%) |  |
| Maternal educational level (n, %) |  |  |  |
| *Elementary school* | 13/38 (34%) | 0 | **<0.001^b^** |
| *High school* | 13/38 (34%) | 2 (4%) |  |
| *Incomplete or complete higher education* | 12/38 (32%) | 46 (96%) |  |
| Monthly family income (n, %) |  |  |  |
| *Less than 3 minimum wages* | 23/27 (85%) | 12/25 (46%) | **0.008^b^** |
| *From 3 to 9 minimum wages* | 4/27 (15%) | 12/25 (46%) |  |
| *More than 9 minimum wages* | 0 | 2/25 (8%) |  |
| ^a^In the case group, some information was not available or was not answered for all mothers. ^b^Chi-squared test or Fisher’s exact test; ^c^Student’s t test or Mann–Whitney U test; Quantitative variables are presented as median and quartiles. | | | |

| **Supplementary Table 2.** Congenital anomalies present in children with CZS. | | | | | | | | | | | | | | | | | | | | | | | | | | | | | | | | | | | | | | | | |
| --- | --- | --- | --- | --- | --- | --- | --- | --- | --- | --- | --- | --- | --- | --- | --- | --- | --- | --- | --- | --- | --- | --- | --- | --- | --- | --- | --- | --- | --- | --- | --- | --- | --- | --- | --- | --- | --- | --- | --- | --- |
|  | **Cases with CZS** | | | | | | | | | | | | | | | | | | | | | | | | | | | | | | | | | | | | | | | |
| **Congenital anomalies** | **1** | **2** | **3** | **4** | **5** | **6** | **7** | **8** | **9** | **10** | **11** | **12** | **13** | **14** | **15** | **16** | **17** | **18** | **19** | **20** | **21** | **22** | **23** | **24** | **25** | **26** | **27** | **28** | **29** | **30** | **31** | **32** | **33** | **34** | **35** | **36** | **37** | **38** | **39** | **40** |
| *Microcephaly* | + | + | + | + | + | + | + | + | + | + | + | + | + | + | + | + | + | + | + | + | + | + | + | + | + | + | + | + | + | + | + | + | + | + | + | + | + | + | + | + |
| *Intracranial calcification* |  | + | + | + | + | + |  |  | + | - | + |  | - |  | + |  |  |  | + |  | + |  |  | + | + | + | + |  |  |  |  | + | + | + | + | + | + | + |  | + |
| *Lissencephaly* |  |  | + | + | + |  |  |  |  |  |  |  |  |  | + |  |  |  |  |  |  |  |  |  |  |  |  |  |  |  |  | + | + | - | - | + | + | + |  | + |
| *Ventriculomegaly* |  |  | + | + | + |  |  |  |  |  |  |  |  |  |  |  |  |  |  |  |  |  |  |  |  |  |  |  |  |  |  | + | - | + | + | - | - | + |  | + |
| *Callosum body alteration* |  |  | + | + | - |  |  |  |  |  |  |  |  |  |  |  |  |  |  |  |  |  |  |  |  |  |  |  |  |  |  | + | + | - | + | + | + | - |  | - |
| *Cerebral atrophy* |  | + | + | + | + |  |  |  |  |  |  |  |  |  |  |  |  |  |  |  |  |  |  |  |  |  |  |  |  |  |  | - | + | + | + | + | + | + |  | + |
| *Other brain anomalies* |  | + | + | + |  |  |  |  |  |  |  |  |  | + |  |  |  |  |  |  |  |  |  |  |  |  |  |  |  |  |  | + |  |  |  |  |  |  |  |  |
| *Ocular alterations* | + | + | - | + | + | + | + | + | + | + | + | + | + | + | + |  |  |  | + |  | - |  |  | - | - | + | - |  | + | - | - | + | - | - | + | + | + | + |  | - |
| ID, patient identifier; (+), Presence of the congenital anomaly in the children; (-), Absence of the congenital anomaly in the children; Blank spaces, Without information. | | | | | | | | | | | | | | | | | | | | | | | | | | | | | | | | | | | | | | | | |

| **Supplementary Table 3**: Analyses of allelic and genotypic frequencies of polymorphisms in TFRC and MIR-124-1 genes in cases according to their characteristics. | | | | | | | | | | | | | | | |
| --- | --- | --- | --- | --- | --- | --- | --- | --- | --- | --- | --- | --- | --- | --- | --- |
| **Characteristic** | ***TFRC* rs406271** | | | | | |  |  | ***MIR-124-1* rs531564** | | | | |  | |
|  | **T** | **C** | **p-value** | **TT**  **(n=23)** | **CT**  **(n=14)** | **CC**  **(n=3)** | **p-value^c^** |  | **C** | **G** | **p-value** | **CG (n=14)** | **GG (n=26)** | **p-value^c^** |  |
| Sex (n, %) |  |  |  |  |  |  |  |  |  |  |  |  |  |  |  |
| *Male* | 35 (58%) | 11 (55%) | 1.000 | 14 (61%) | 7 (50%) | 2 (67%) | 0.883 |  | 8 (57%) | 38 (58%) | 1.000 | 8 (57%) | 15 (58%) | 1.000 |  |
| *Female* | 25 (42%) | 9 (45%) |  | 9 (39%) | 7 (50%) | 1 (33%) |  |  | 6 (43%) | 28 (42%) |  | 6 (43%) | 11 (42%) |  |  |
| Ethnicity (n, %) |  |  |  |  |  |  |  |  |  |  |  |  |  |  |  |
| *Black* | 45 (75%) | 17 (85%) | 0.573 | 17 (64%) | 11 (79%) | 3 (100%) | 0.951 |  | 2 (14%) | 16 (24%) | 0.796 | 2 (14%) | 7 (27%) | 0.749 |  |
| *White* | 15 (25%) | 3 (15%) |  | 6 (26%) | 3 (21%) | 0 (0%) |  |  | 12 (86%) | 50 (76%) |  | 12 (86%) | 19 (73%) |  |  |
| Trimester of ZIKV infection (n, %)^a^ |  |  |  |  |  |  |  |  |  |  |  |  |  |  |  |
| *1st* | 38 (84%) | 10 (66%) | 0.076 | 15 (88%) | 8 (73%) | 1 (50%) | 0.166 |  | 7 (70%) | 41 (82%) | 0.520 | 7 (70%) | 17 (85%) | 0.487 |  |
| *2nd* | 6 (13%) | 2 (13%) |  | 2 (12%) | 2 (18%) | 0 (0%) |  |  | 2 (20%) | 6 (12%) |  | 2 (20%) | 2 (10%) |  |  |
| *3rd* | 1 (3%) | 3 (2%) |  | 0 (0%) | 1 (9%) | 1 (50%) |  |  | 1 (10%) | 3 (6%) |  | 1 (10%) | 1 (5%) |  |  |
| Congenital anomalies (n, %)^b^ |  |  |  |  |  |  |  |  |  |  |  |  |  |  |  |
| *Multiple* | 44 (73%) | 16 (80%) | 0.767 | 17 (74%) | 10 (71%) | 3 (100%) | 0.868 |  | 9 (64%) | 51 (77%) | 0.499 | 9 (64%) | 21 (81%) | 0.446 |  |
| *Isolated* | 16 (27%) | 4 (20%) |  | 6 (26%) | 4 (29%) | 0 (0%) |  |  | 5 (36%) | 15 (23%) |  | 5 (36%) | 5 (19%) |  |  |
| ^a^Some information was not available or not provided by all mothers. ^b^Note that imaging exam results were not available for cases classified as isolated congenital anomalies. ^c^Chi-squared test or Fisher’s exact test. | | | | | | | | | | | | | | | |
